# A Screen of Autophagy Compounds Implicates the Proteasome in Mammalian Aminoglycoside-Induced Hair Cell Damage

**DOI:** 10.3389/fcell.2021.762751

**Published:** 2021-10-26

**Authors:** Clara Draf, Taylor Wyrick, Eduardo Chavez, Kwang Pak, Arwa Kurabi, Anke Leichtle, Stefan Dazert, Allen F. Ryan

**Affiliations:** ^1^Department of Surgery/Otolaryngology, University of California, San Diego, San Diego, CA, United States; ^2^Department of Otolaryngology, St. Elisabeth-Hospital, Ruhr University Bochum, Bochum, Germany; ^3^Department of Biology, University of California, San Diego, San Diego, CA, United States; ^4^Department of Otolaryngology, University Medical Center Schleswig-Holstein, Lübeck, Germany; ^5^Department of Neurosciences, University of California, San Diego, San Diego, CA, United States; ^6^VA San Diego Healthcare System, San Diego, CA, United States

**Keywords:** autophagy, hair cell, aminoglycoside–ototoxicity, *in vitro* screen, inner ear

## Abstract

**Introduction:** Autophagy is a degradative pathway to safely break down and recycle dysfunctional cellular components. There is prior evidence of autophagy participation during hair cell (HC) damage. Our goal was to screen compounds targeting different aspects of autophagy for their effects on HC loss due to an ototoxic aminoglycoside, gentamicin (GM).

**Methods:** The SELLECKChem autophagy compound library, consisting of 154 compounds with defined autophagy inducing or inhibitory activity, was used for targeted screening *in vitro* model of ototoxicity. Organ of Corti from postnatal days 3–5 pou4f3/GFP transgenic mice (HCs express green fluorescent protein) were utilized. The organs were micro-dissected, and basal and middle turns divided into micro-explants individually placed into the single wells of a 96-well plate. Samples were treated with 200 μM of GM plus three dosages of tested compound and cultured for 72 h. Negative controls were treated with media only; positive ototoxicity controls were treated with GM only.

**Results:** The majority of the library compounds had no effect on GM-induced HC loss. However, 18 compounds exhibited a significant, protective effect, two compounds were protective at low dosage but showed enhanced GM toxicity at higher doses and one compound was toxic to HCs in the absence of GM.

**Conclusions:** This study evaluated many autophagy compounds that have not been tested previously on HCs. The disparate results obtained underscore the complexity of autophagy events that can influence HC responses to aminoglycosides, but also implicate the proteosome as an important damage mechanism. The screening results can serve as basis for further studies with protective compounds as potential drug targets.

## Introduction

Ototoxicity is a significant side effect of some valuable medications that are used to treat life-threatening diseases. Ototoxic drugs can cause irreversible damage to the inner ear, leading to loss of hearing and balance function. A major class of ototoxic drugs are the aminoglycoside antibiotics. Aminoglycoside-induced ototoxicity occurs in as many as 50% of patients with multi-drug resistance tuberculosis and in up to 20% of children treated for cystic fibrosis (Al-Malky et al., 2015; Sagwa et al., 2015).

The aminoglycoside antibiotic, gentamicin (GM) was discovered in 1963 as an extract of *Micromonospora purpurea*. It is recommended as an effective, safe and essential medicine (Abou-Zeid et al., 1978; Obregon et al., 1994), widely used to treat endocarditis, meningitis, pelvic inflammatory disease, urinary tract infections, bone infections, pneumonia and other bacterial infections, including sepsis (Hamilton, 2001). Although GM is an extremely effective antimicrobial, ototoxicity is its major side effect (Kim et al., 2017; Sapizhak et al., 2017). The sensory hair cells (HCs) of the inner ear are major targets of GM toxicity. As terminally differentiated cells, HCs are unable to regenerate. Loss of HCs due to ototoxicity causes permanently reduced hearing sensitivity, with complete deafness in severe cases (Xing et al., 2017).

Oxidative stress and apoptosis are major drivers of GM induced ototoxicity, which implies that autophagy is also involved (He et al., 2018). Autophagy is an intracellular, lysosomal-mediated process in which dysfunctional cell material is broken down and recycled (Mizushima, 2011; He et al., 2018). This results in a dynamic relationship between autophagy, oxidative stress, damage to organelles and proteins, and apoptosis (Lee et al., 2012; Benkafadar et al., 2019). Autophagy is an important component of apoptosis, packaging damaged cellular constituents prior to cell dissolution. Autophagy can also play a role in the survival of cells in stress conditions, such as starvation and oxidative stress, by preventing the intracellular and extracellular spread of damage (Mizushima et al., 2008; Esclatine et al., 2009; Rabinowitz and White, 2010). Autophagy participates in regulating other physiological processes in non-stress situations, such as cell proliferation and differentiation, where cellular constituents must be destroyed or recycled (Esclatine et al., 2009; Oh et al., 2012; Magarinos et al., 2017). However, when autophagy is over-enhanced, cellular components essential for cell survival can be degraded, leading to cell death (Chen et al., 2010). Given its multifunctional nature, it is not surprising that autophagy dysfunction has been suggested to be involved in several pathological changes, such as cancer, inflammation, neurodegenerative disease, and metabolic disorders (Levine and Kroemer, 2008; Arroyo et al., 2014; Ryter et al., 2014).

**FIGURE 1:**
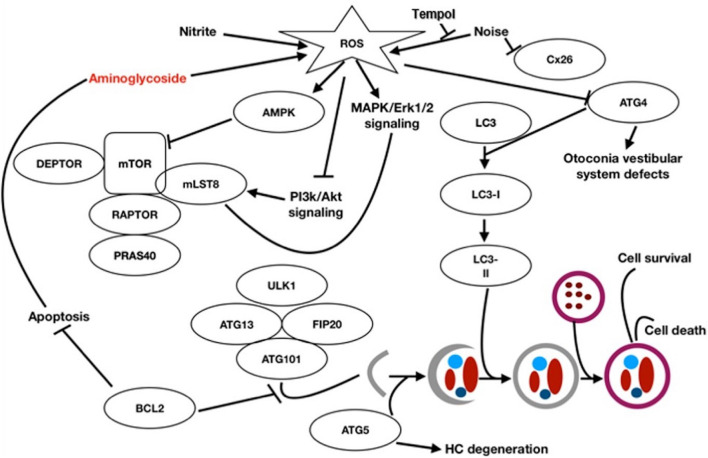
Autophagy signal pathway and relationship with the auditory system [Figure modified after He et al. (2018)] Macroautophagy: A part of the endoplasmic reticulum encloses the structures to be degraded and isolates them with a double membrane (autophagosome). The autophagosome binds to a lysosome under hydrofusion. This results in the degradation of the autophagosomal components.

Autophagy (Figure 1) is initiated by the formation of double membrane cytosolic vesicles, called the phagophore or isolation membrane, that contains damaged cellular constituents (Mizushima and Komatsu, 2011). The phagophore matures into a closed double-membrane-bound structure and then fuses with lysosomes, called the autophagosome, for disposal of its contents (Ohsumi, 2001). Autophagy involves the coordinated, and sequential activation of specific sets of autophagy-related genes (Ohsumi, 2001).

In the inner ear, autophagy has been shown to be an essential catabolic mechanism which is necessary for normal embryonic development (Taylor and Kirkegaard, 2008). The mechanism also responds to otic injury in the adult mouse, for autophagic recycling of intracellular components and elimination of deleterious molecules and organelles (Taylor and Kirkegaard, 2008). Inhibition of the autophagic pathway has been shown to provoke HC degeneration, the damage of neurogenesis and the aberrant axonal outgrowth, leading to hearing loss (Aburto et al., 2012). Conversely, activation of autophagy in HCs and oC-derived cell lines reduces ROS levels and also promotes cell survival (He et al., 2018). With respect to aminoglycoside ototoxicity, a relatively small number of autophagy studies have been performed. However, inhibition of autophagy has been shown to enhance Neomycin damage to HCs (He et al., 2018), and impaired autophagy has been proposed as a mechanism of delayed HC death in GM ototoxicity (Kim et al., 2017).

Given the complexity of the autophagy process and its diverse roles in cellular function and damage, the objective of this study was to screen compounds targeting different aspects of autophagy for their effects on HC loss due to GM. The goal was to enhance understanding of the role of autophagy in ototoxicity, and to identify and compare potentially protective compounds.

## Materials and Methods

### Animals

Transgenic neonatal mice (3–5 days) in which eGFP was selectively expressed in HCs under the control of a *pou4f3* promoter construct (Masuda et al., 2011) were used. All experiments were performed to National Institutes of Health guidelines and approved by the Institutional Animal Care and Use Committee of the VA San Diego Medical Center.

### Microdissection

**FIGURE 2:**
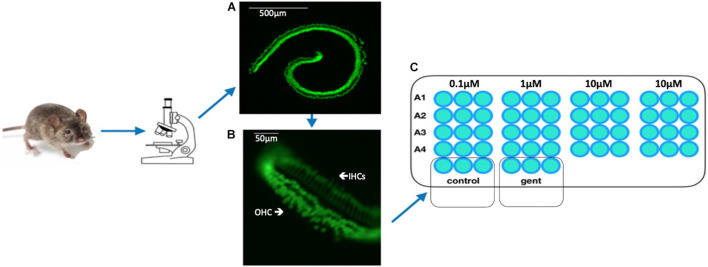
Schematic of workflow from the organ of Corti **(A)** of the pou4f3/GFP transgenic mouse to microexplants **(B)** into the 96-well plate which contained nutrient medium, autophagy compound (A1–A4) and GM **(C)**. The microexplant **(B)** at the beginning of the treatment. The typical structure of one row of IHCs and 3 rows of OHCs is visible.

The organ of Corti (oC) was dissected from the cochlea of 3–5 postnatal day *pou4f3*/eGFP mouse neonates. The apical region of each epithelium, which is relatively insensitive to aminoglycoside toxicity, was discarded. The basal and middle regions of the epithelium were divided using a diamond scalpel into micro-explants consisting of approximately 20 inner HCs and the 60 associated outer HCs. Micro-explants were individually plated in each well of a flat-bottom 96 well plate in media consisting of DMEM F-12(Gibco) plus 30 U/ml Penicillin and 5% FBS, and maintained in a humidified tissue culture incubator (37°C, 5% CO_2_). Methods workflow is depicted in Figure 2. Mature hair cells at later times are more difficult to micro-dissect and also do not survive well in culture.

### Screening

**FIGURE 3:**
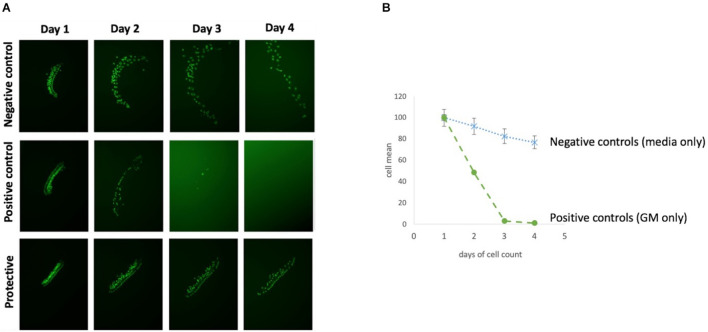
Overview of HC decrease over 72 h. **(A)** Micro-explant pictures showing the fluorescent HCs over the experimental time course and **(B)** Illustration survival curves of positive and negative control of an autophagy compound.

Targeted screening was performed using the SelleckChem Autophagy Compound Library (Z167594, Selleck Chem, Houston, TX, United States), consisting of 154 autophagy-related compounds targeting the: PI3K/Akt/mTOR (15%), DNA damage and sirtuin (15%), cell cycle (15%), proteasome and ubiquitin (10%), transmembrane transporters (10%), apoptosis (5%), cytoskeletal signaling (5%), GPCR and G-protein (5%) among other (20%) pathways. The library represents a variety of classes of compounds, including a number of autophagy inducing and autophagy inhibiting compounds which at present had not previously been applied to HC damage. Test compounds were initially dissolved in DMSO and diluted in culture media with the total amount of DMSO adjusted to a final concentration of 0.1%. Each experimental oC micro-explant was pretreated for 24 h with one of the library compounds at concentration of 0.1, 1, or 10 μM, performed in triplicate wells. On the following day media containing 200 μM gentamicin as well as the test autophagy compound with the appropriate concentration was added, and the micro-explants were cultured for 72 h. Untreated (negative) controls were maintained in media alone and positive controls were treated with 200 μM GM alone (Figure 3). Compound controls were treated with the highest concentration of the compound (10 μM) alone. Media for all control groups contained 0.1% DMSO, to match the experimental groups. Compounds were screened in duplicated 96-well plates with 5–7 autophagy compounds per plate, plus controls. Green fluorescent protein-positive HCs were imaged by fluorescence microcopy on each day of treatment, and survival curves were generated for each compound and condition. HC counts, including both inner and outer HCs, were evaluated in ImageJ with the Macro “ITCN” or “Cell Counter,” and normalized as percentages to the number of HCs present on D1, prior to the start of GM treatment. Any micro-explants that did not attach and flatten in the well by D1 were excluded, because HC counts could not be accurately quantified at that time. There were sufficient wells on each plate that three micro-explants per condition could almost always be accommodated even with some unattached samples. Autophagy compounds that showed a significant protective effect, as compared to the control, in the initial screen were repeated for a second round at the three different concentrations. A compound was scored as “protective” if there was an overall significance for six wells of any concentration. A dose-dependent protection was attributed if there was a statistical significance at all three concentrations (18 wells).

Statistical analysis was performed using GraphPad Prism6, StatView5, using the Kruskal–Wallis non-parametric ANOVA to detect treatment effects. Individual condition comparisons were performed using the Mann–Whitney *U* test, with correction for multiple comparisons. For purposes of the figures, standard deviations were calculated from the non-normalized HC counts. Validation Redox compound “hits” were identified in the initial round of screening as deviating significantly from the controls. Following this initial screen, repeat plates were prepared in an identical manner for all hits, for a total N of six micro-explants. Statistical analysis was then repeated. Compounds that demonstrated a significant effect when the following round was included were considered to be confirmed.

## Results

### Control Micro-Explants and Gentamicin Cytotoxicity

Each 96 well plate had its own negative and positive control. HC counts from negative and positive control micro-explants were converted to percent survival relative to Day 1 (D1, just prior to GM exposure), and averaged across all plates. Imaging of GFP-positive HCs in control wells typically showed HC survival similar to that illustrated in Figure 3. Untreated (negative control) micro-explants maintained only in culture media showed near-complete HC survival from D1-D3 ranging from 94% on D2 to 91% on D3. On Day 4, HC survival was reduced to a range of 80–68%. In contrast, HCs treated with 200 μM GM (positive control) showed significant losses by D2 with an average HC survival of 52%, D3 HC survival was 13%, while D4 showed continued losses to 8% survival. As expected, Kruskal–Wallis and Mann–Whitney analysis showed a significant difference between negative and positive controls from D2–D4. No differences were observed between explants from the basal or middle regions of the cochlea, consistent with the high dosage of GM. Nevertheless, there were plates in which some variation occurred in the positive and/or negative controls. Therefore the results for each compound were compared statistically to positive controls from the same plate, generated with the same batches of media and GM. Additionally, each compound that was statistically different from the control was repeated for validation.

### Library Results

The effects of the 154 compounds could be divided into three groups, autophagy compounds which exhibited:

I. No effect.
II. Protective effect.
III. Toxic effect.

#### Group I: No Effect

**FIGURE 4:**
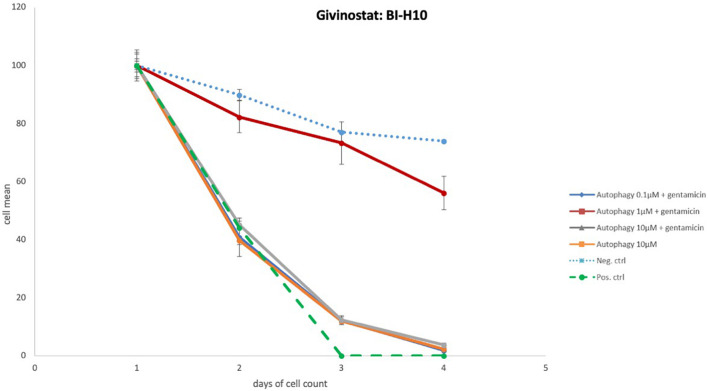
Autophagy compound without protective effects. Illustration of Survival curves of representative compound that had no effect on GM-treated oC explants. Treatment with the highest concentration (10 μM) of autophagy compound resulted in HC numbers similar to that seen for negative control micro-explants cultures in media alone. Treatment with compound plus GM was not significantly different from gentamicin alone.

The majority of the 154 autophagy- inducing and/or -inhibiting compounds had no significant effect on HCs after GM treatment. An example is illustrated in Figure 4. When the highest concentration (10 μM) of autophagy compound was added to the micro-explant HC numbers were similar to that seen for negative control micro-explants cultures in media alone. Adding three different concentrations of autophagy compound plus 200 μM GM produced HC survival rates comparable to those observed with GM alone.

#### Group II: Protective

**FIGURE 5:**
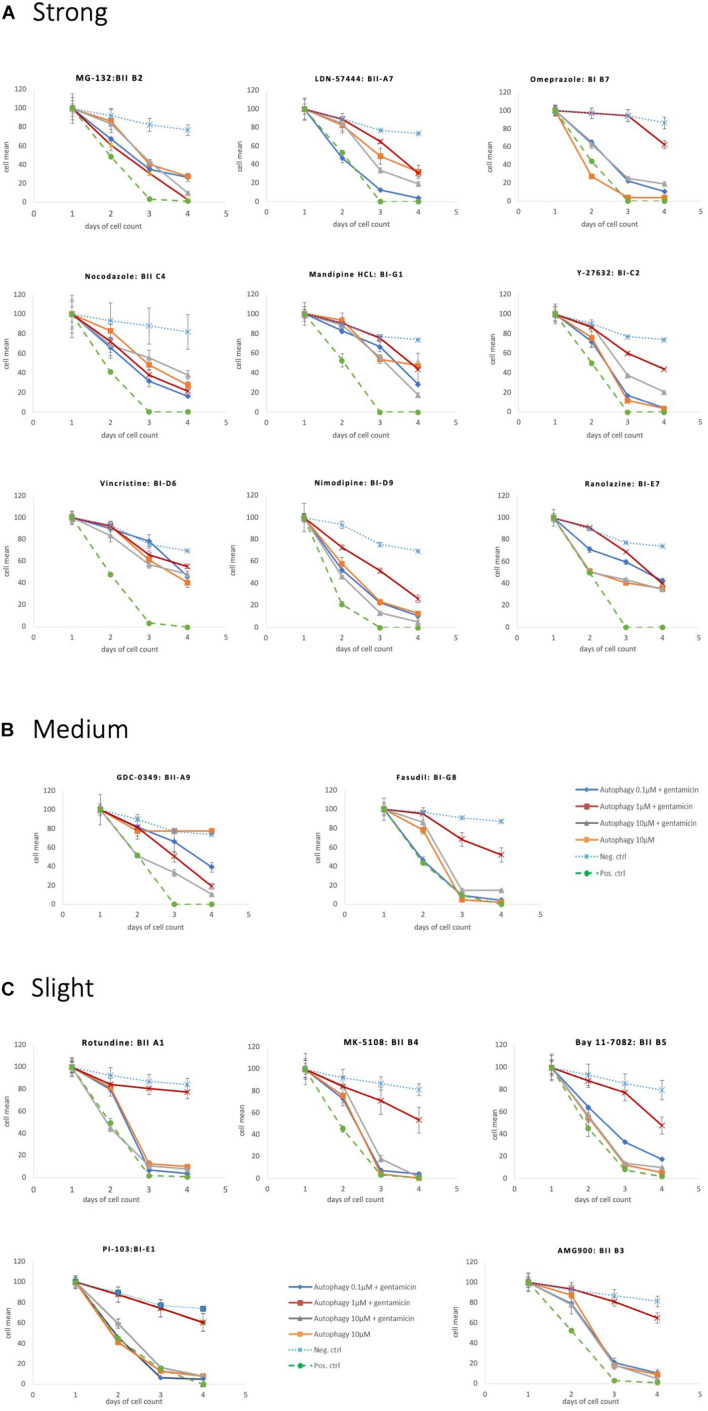
Protective autophagy compounds Survival plots showing the autophagy compounds classified as **(A)** Strong, **(B)** medium and **(C)** slight depending on the HCs survival curves.

**FIGURE 6:**
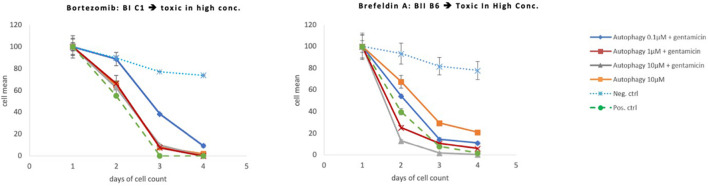
Protective and toxic autophagy compounds. These compounds had a strong protective effects at low doses but were toxic at the highest concentration dose tested (10 μM).

Eighteen autophagy compounds showed statistically significant protection of HCs in micro-explants treated with both the compound and gentamicin (Table 1). Within the group of protective compounds the observed protective effect could be divided into strong, medium and slight protection (Table 2). Strong protective compounds exhibited significant HC survival on all 3 days, varying in concentration (Figure 5A). Medium protective compounds showed improved HC survival on 2 days with one or two concentrations (Figure 5B), while slightly/mildly protective compounds reduced HC loss on D1 and only for 10 μM concentration (Figure 5C). There were two test substances that exhibited a bimodal trait i.e., significant protection at low concentrations, but enhancing GM damage at the highest concentration. Bortezomib and Brefeldin A exhibited significant protective traits at 0.1 μM, Brefeldin A also with 1 μM on Day 2, they both demonstrated significantly enhanced early HC loss due to gentamicin at 10 μM (Figure 6). We observed this “push-pull” effect with tyrosine kinase inhibitors in previous studies (Ryals et al., 2017).

**TABLE 1:** Overview of the results of the SELLECKChem autophagy compound library.

Compounds tested	No effect	Toxic effect	Protective effect
*N* = 154 = 100%	135 = 87.7%	1 = 0.6%	18 = 11.7%

**TABLE 2:** Overview of the protective autophagy compounds identified in the *in vitro* screening assay.

Strong = day 2 + 3 + 4		Medium = day 2 + 3		Weak = day 2	
Compound	Function	Compound	Function	Compound	Function
MG-132 (AMG-IIB2)	Protein homeostasis inhibitor	Fasudil (AMG-IG8)	Microtubule interfering agents	Rotundine (AMG-IIA1)	Calcium regulation
Omeprazole (AMG-IB7)	Protein homeostasis inhibitor	GDC-0349: (AMG-IIA9)	Inhibitors of the PI3K/AKT/mTOR pathway	BAY 11-7082 (AMG-IIB5)	Inflammation and immunity inhibitor
Bortezomib: (AMG-IC1)	Protein homeostasis inhibitor			AMG-900: (AMG-IIB3)	Cell cycle regulator
LDN-57444: (AMG-IIA7)	Protein homeostasis inhibitor			PI-103 (AMG-IE1)	Inhibitors of the PI3K/AKT/mTOR pathway
Vincristine: (AMG-ID6)	Microtubule interfering agents			MK-5108: (AMG-IIB4)	Cell cycle regulator
Nocodazole: (AMG-IIC4)	Microtubule interfering agents				
Y-27632:(AMG-IC2)	Microtubule interfering agents				
Manidipine HCL (AMG-IIG1)	Calcium regulation				
Nimodipine: (AMG-ID9)	Calcium regulation				
Brefeldin A: (AMG-IIB6)	Cell cycle regulator				
Ranolazine (AMG-IE7)	Potassium channel blocker				

#### Group III: Toxic

**FIGURE 7:**
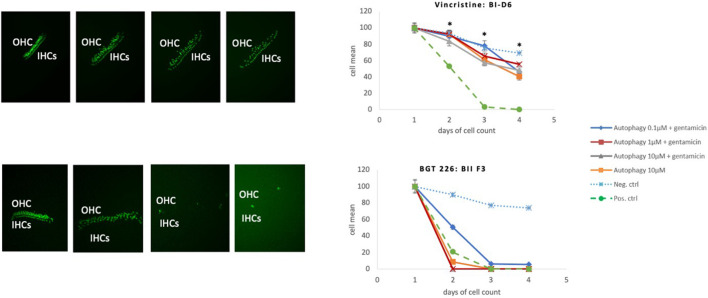
Toxic autophagy compounds. Comparison of an autophagy compounds with a protective effect (Vincristine) with an autophagy compound with a toxic effect (BGT 226). Protective Effect: Vincristine has a significant protective effect in all three dosages *(*p* < 0.05). Toxic effect of BGT 226 (AMG-IIF3), Inhibitor of the PI3K/AKT/mTOR pathway, exhibiting HC loss even in the absence of GM.

One autophagy compound BGT 226 showed HC decrease when treated with 10 μM of the compound alone (even in the absence of gentamicin). This was significant at 48 and 72 h (Figure 7).

## Discussion

### Summary

Using an assay based on micro-explants of the neonatal mouse organ of Corti, a variety of autophagy compounds were screened for their ability to alter aminoglycoside damage to mammalian cochlear HCs *in vitro*. All statistically significant “hits” were confirmed by re-screening. Of a total of 154 compounds tested, 18 exhibited protective effects against GM toxicity, two of these compounds: Bortezomib, a protein homeostasis inhibitor and Brefeldin A, a cell cycle regulator were protective at low dosage but showed enhanced GM toxicity at high dosage. One compound, BGT 226 an Inhibitor of the PI3K/AKT/mTOR pathway was toxic to HCs in the absence of GM.

### HC Protective Autophagy Compounds

Among the 154 compounds tested, 18 proved to be protective to HCs. To our knowledge, none of the autophagy compounds identified in the screen have been previously identified as influencing HC damage *in vitro* or *in vivo*. While all exhibited statistically significant effects, the range of protection varied from slight to strong. The protective autophagy compounds had a broad spectrum of activities ranging from protein homeostasis inhibitors, cell cycle and receptor kinase inhibitors to calcium channel and calcium uptake blockers. However, unlike our prior screens of kinase inhibitors (Ryals et al., 2017) and antioxidants (Noack et al., 2017), many of the compounds in the autophagy library are not highly specific to autophagy. The protective autophagy compounds found in this screen can be divided into the following categories, listed in order of the number of protective library compounds per category.

#### Inhibitors of Protein Homeostasis

Proteasomes are protein complexes that degrade unneeded, misfolded or damaged proteins by proteolysis. Misfolded proteins trafficked from the Golgi to the ER are retained in the ER and retro-translocated to the cytosol in vesicles (Omari et al., 2018). Along with damaged proteins, they are tagged for proteasome proteolysis by ubiquitins (Taxis et al., 2002). Autophagy is thought to co-operate with the ubiquitin-proteasome system in responding to cell injury (Ji and Kwon, 2017). The proteasome is known to protect cells from degeneration by removing damaged proteins (Jones and Tepe, 2019), but an overactive proteasome can enhance tissue damage (Meller, 2009; Chen et al., 2010). Six compounds out of the 18 protective compounds were proteasome inhibitors.

a. LDN-57444: Reversible, competitive proteasome inhibitor for Uch-L1.
b. MG-132: Cell permeable proteasome and calpain inhibitor, in low dosage protective, in high dosage toxic.
c. Bortezomib: Proteasome inhibitor.
d. Omeprazole: Proton pump inhibitor. Proton pump inhibition reduces the expression of proteasome subunits, leading to proteasome inhibition (Cao et al., 2020).
e. PYR-41: Ubiquitin Proteasome inhibitor. Reduces ubiquitination, required for proteasome degradation of proteins, but also inhibits NFkB depending on its half-maximal inhibitory concentrations (IC50) which has been identified between 10–25 μM in cells (Yang et al., 2007).
f. Brefeldin A. An inhibitor of macro-autophagy, reduces the release of proteins from the ER.

The library had twelve inhibitory compounds targeting the proteasome pathway and of which six were identified protective in our screen. These results strongly suggest that the proteasome overactivity contributes to HC damage. Cheng et al. (2016) identified that Atoh1 (Atonal BHLH Transcription Factor 1) was regulated by the ubiquitin-proteasome system and hence ubiquitin proteasome pathway appears necessary for hair cell fate determination and survival. Also, we recently reported that multiple proteins of the ubiquitin-proteasome system are up-regulated in the cochlea following noise damage (Jongkamonwiwat et al., 2020), suggesting the possibility of a similar involvement in noise-induced HC damage associated with protein abundance and protein misfolding.

#### Microtubule Interfering Agents

In autophagy, mature autophagosomes are transported to lysosomes along microtubules with force generated by the actin cytoskeleton. Microtubules are also involved in the transport of other cellular constituents, cell structure and cell migration. The library had twelve compounds associated with autophagy, microtubules and actin of which four inhibitors of were protective.

a. Nocodazole: Reversible inhibitor of Abl and microtubule polymerization:
b. Vincristine: binds tubulin protein and counteracts the formation of microtubules.
c. Y- 27632: Inhibitor of ROCK-1, which promotes contractile force generation in the actin cytoskeleton and movement along microtubules.
d. Fasudil: ROCK inhibitor.

#### Inhibitors of Calcium Regulation

Calcium regulates autophagy at several different points in the process (Bootman et al., 2018). Increases in intracellular calcium, from calcium channels, calcium uptake or release from mitochondria, can induce autophagy. Conversely, calcium release leading to entry into mitochondria increases ATP production, which inhibits mitochondria (Kondratskyi et al., 2013). However, calcium also plays multiple additional roles in cells. Loss of calcium homeostasis is a major factor in cell injury and death through multiple mechanisms include apoptosis and necrosis (Dong et al., 2006). Intracellular calcium is tightly regulated in part to prevent the accumulation of excessive levels. HCs in particular contain high levels of calcium buffering proteins and express multiple calcium channels and transporters. Calcium subserves multiple normal functions of HCs (Heller et al., 2002). A number of studies have highlighted the role of calcium in HC damage, including release of calcium from the endoplasmic reticulum and mitochondria. It has been suggested that differences in HC vulnerability to damage along the length of the cochlea are related to variation in the ability to handle calcium load (Fettiplace and Nam, 2019). It has been suggested that calcium channel blockers should be investigated as therapeutic agents for acquired hearing loss (Naples, 2017). In this screen, the library had fourteen compounds that influence intracellular calcium levels and four were found to be protective.

a. Manidipine: Calcium channel blocker (L and T gated channels).
b. Nimodipine: Calcium channel blocker (L gated channels).
c. Ranolazine: Calcium uptake inhibitor.
d. Rotundine: Rotundine is a natural plant alkaloid with complex effects. It has been reported to block L-type calcium channels, but also dopamine receptors, and to activates potassium channels. It has been shown to be neuroprotective e.g., in alpha synuclein accumulating diseases such as Parkinson (Ghanem et al., 2021).

The expected results of these inhibitors would be to reduce intracellular calcium. While this strongly supports a critical role for calcium in HC damage, it cannot be attributed with any certainty to the regulation of autophagy.

#### Inhibitors of the PI3K/AKT/mTOR Pathway

The PI3K/AKT/mTOR is an evolutionally conserved cascade that integrates signals from multiple pathways, including nutrients (e.g., amino acids and glucose), growth factors (e.g., EGF, PDGF, IGF-1), hormones (e.g., leptin), and stresses (e.g., starvation, hypoxia, and DNA damage) to regulate a wide variety of eukaryotic cellular functions, such as autophagy, translation, transcription, protein turnover, metabolism, energy balance, and stress response, as well as cell proliferation, growth, differentiation and survival. mTOR can also be activated independently of PI3K and AKT. The library contains 25 compounds related to PI3K/mTOR and three inhibitors were protective.

a. PI-103: PI3K/mTOR inhibitor.
b. BGT 226: PI3K/mTOR inhibitor.
c. GDC-0349: Selective ATP-competitive inhibitor of mTOR.

Because of the diverse rolls of the PI3K/AKT/mTOR pathway, the mechanism by which these inhibitors protect HCs is not clear. mTOR reduces autophagy by negatively regulating several key molecules (Kim and Guan, 2015), so the promotion of autophagy is one possibility. However, in our prior screen of kinase inhibitors (Ryals et al., 2017), one EGFR inhibitor, two PDGF receptor and two AKT inhibitors were found to be protective. This result is consistent with a damaging role for the PI3K/AKT/mTOR pathway, which is activated by growth factors. However, one of the autophagy compounds, BGT 226, showed a “push pull” relationship with a toxic effect in the highest concentration leading to HC death. Deletion of either AKT1 or AKT2 has been shown to enhance HC death due to aminoglycosides (Brand et al., 2015). It is possible that the three PI3K/mTOR inhibitors preferentially target PI3K/mTOR mediation of stress responses. Alternatively, they may target one of the several mTOR responses that are independent of AKT.

#### Inhibitors of the Cell Cycle

Aurora kinase A is critical to the process of mitotic spindle formation and separation, and thus of cell division. Inhibition of Aurora kinase A blocks cell proliferation and can induce autophagy (Zou et al., 2012). In our screening assay two inhibitors of Aurora kinase inhibitors showed a protective effect:

a. AMG900: Selective pan-Aurora kinase inhibitor.
b. MK-5108: Selective Aurora kinase A inhibitor.

Overall the library had 20 compounds related to the Aurora kinase pathway and only two compounds were protective. This is consistent with the finding that maintaining the fully differentiated state is critical for HC survival and that forced cell cycle re-entry in differentiated, post-mitotic cells in response to stress can lead to mitotic catastrophe and cell death (Weber et al., 2008; Sharma et al., 2017). This provides strong evidence that one aspect of gentamicin-induced HC damage may be induction of the cell cycle.

#### Inhibitor of Inflammation and Immunity

NFkB Inhibitor: BAY 11-7082. The role of immune responses and inflammation in HC damage is increasingly recognized (Frye et al., 2019). Damage to HCs results in the release of inflammatory mediators such as the inflammatory cytokine TNFα (Tan et al., 2016). Damaged cells can also release cellular constituents that serve as damage-associated molecular patterns (DAMPs). Damps in turn can activate innate immune receptors such as the Toll-like receptors (TLRs), leading to the production of inflammatory cytokines such as IL1β and TNFα, as well chemokines that attract immune cells including macrophages. Many inflammatory responses are mediated by the transcription factor NFkB, which can also stimulate autophagy. An inhibitor of this factor was protective in the screen. This result supports a role for inflammation in ototoxic HC damage.

#### Protein Trafficking

An inhibitor of protein trafficking was protective, Brefeldin A. Brefeldin A is an inhibitor of protein trafficking between the endoplasmic reticulum and the Golgi apparatus (Chardin and McCormick, 1999). These results suggest that the movement of intracellular elements, possibly including autophagosomes, contribute to ototoxic HC damage.

The majority of the compounds that proved to be protective to HCs against gentamicin damage would be expected to reduce autophagy. While several of these compounds have complex effects, the fact that multiple autophagy inhibitors reduced HC loss argues for an autophagy role in promoting HC damage due to GM. Levano and Bodmer found that mutation of the *Stat1* gene protected HCs from ototoxic damage in part by modulating autophagy genes so as to reduce autophagy (Levano and Bodmer, 2015).

Autophagy is often considered to be protective of cells under stress, but it has been shown that excessive activation of autophagy can lead to autophagic cell death, or autosis (Liu and Levine, 2015). The high dosage of GM used in this study may have induced excessive autophagy. Apoptosis, necrosis and necroptosis have been shown to participate in HC death (Ruhl et al., 2019). However, autosis has not previously been linked to HC damage. Autosis is characterized by nuclear shrinkage with focal concavities and ballooning of the perinuclear space. These characteristics are visible in some published electron micrographs of aminoglycoside-damaged HCs (Lang and Liu, 1997).

While most autophagy inhibiting compounds were protective, we also observed protection with compounds that would be expected to enhance autophagy. Again, while these compounds have complex effects, the results support the concept that autophagy can be protective against aminoglycoside damage. It seems likely that autophagy plays a complex role in ototoxic HC damage, such that manipulation of different aspects of the process can have differing effects. The interactions of identified compounds with the autophagy machinery warrants further mechanistic studies because these candidate leads may employ one or more of several mechanisms. Therefore, differences in their effectiveness may vary with differences in the cellular processes involved and aminoglycoside impact. Given the complexity, it would not be expected that all compounds in a category would have equivalent effects on cell damage and survival.

### Mammalian Organ of Corti Micro-Explant Assay

There are multiple screening assays available today. The assay presented here, like many preclinical assays, has both advantages and disadvantages. A major advantage is that organ of Corti micro-explant screening employs mammalian cochlear HCs. Cochlear HCs in mammals include inner and outer HCs, as in humans, while the HCs of other classes of non-mammalian animals are quite different from human HCs. This is important, since outer HCs are more sensitive to damage than other HC types. They are also highly specialized, with structural and functional features that are very distinct. The damage process in these highly specialized cells may not be adequately modeled by that in other HC types, or by those in immortalized cell lines derived from the organ of Corti. These cell lines are de-differentiated, and express genes of both early developing HCs and supporting cells.

In general, a larger number of compounds can be tested in an *in vitro* oC assay than can be achieved using *in vivo* models, which are the only alternatives that allows testing of mammalian cochlear HCs. For this assay, the use of oC micro-explants enables an even larger number of compounds evaluations. Several micro-explants can be generated from each murine organ of Corti. Moreover, because the HCs of *pou4f3*/GFP mice are selectively fluorescent, they do not need to be stained and can be quantified throughout the period of culture in a single micro-explant. Thus multiple groups of explants are not necessary to determine the kinetics of compound effects. Because of these features, screening of a few hundred compounds is readily achieved. The ototoxin chosen for the assay, GM, is commonly prescribed for life-threatening infections and is thus relevant to human therapy.

Disadvantages include the fact that the assay is based on neonatal HCs, which have known differences from adult HCs (Hur et al., 2018). Mature adult HC do not survive in culture and many studies have demonstrated that cell cycle reactivation in mammalian cochlear HCs results in cell death *in vitro* and *in vivo* (Laine et al., 2007; Sulg et al., 2010). Moreover, like in other screening techniques, the micro-explant screening assay identified candidates must be confirmed by post-screen characterization of hits and testing in *in vivo.* Finally, when compared to immortalized cell line or zebrafish lateral line screens, the assay is only medium-throughput. Very large compound libraries cannot be screened, and the number of compound and ototoxin dosages tested must be limited.

## Conclusion

The preponderance of the results from our screen of an autophagy library suggest that autophagy contributes to ototoxic HC damage in a complex manner. However, the compounds tested have many potential alternative effects. For this reason we cannot conclude with confidence that changes in autophagy were the primary reason for HC protection. Additional studies will need to be performed to further validate the results of our screen and to determine the relationship between autophagy and HC damage *in vivo*. However, the data strongly implicate the proteasome and proteolysis as contributors to HC damage.

## Data Availability Statement

The original contributions presented in the study are included in the article/Supplementary Material, further inquiries can be directed to the corresponding author/s.

## Ethics Statement

The animal study was reviewed and approved by VA IACUC.

## Author Contributions

CD, TW, EC, and KP performed the laboratory experiments and screening assay. CD, AK, and AL analyzed the data. CD, SD, and AR conceived the study and designed the experiments. CD wrote the initial manuscript and generated figures. All authors reviewed and approved the manuscript.

## Conflict of Interest

AR is a co-founder, shareholder, and consultant of Otonomy Inc., which develops slow-release drug compounds for the treatment of middle and inner ear diseases. The UCSD Committee on Conflict of Interest has approved this relationship. Otonomy Inc., played no part in the research reported here. The remaining authors declare that the research was conducted in the absence of any commercial or financial relationships that could be construed as a potential conflict of interest.

## Publisher’s Note

All claims expressed in this article are solely those of the authors and do not necessarily represent those of their affiliated organizations, or those of the publisher, the editors and the reviewers. Any product that may be evaluated in this article, or claim that may be made by its manufacturer, is not guaranteed or endorsed by the publisher.
