# Supplementary material for: A Screen of Autophagy Compounds Implicates the Proteasome in Mammalian Aminoglycoside-Induced Hair Cell Damage
Source: Front Cell Dev Biol. 2021 Oct 26;9:762751. doi: 10.3389/fcell.2021.762751 (PMC8576371; doi:10.3389/fcell.2021.762751)
Supplement: Supplementary file 1 [file Table_1.DOCX]

**Supplementary Table 1. The SELLECKChem autophagy compound library AMG I and II.**

**Library AMG I**

IA1 ABT-737

IA2 Varinostat

IA3 LAQ 824

IA4 SRT 1720

IA5 Valproic aid

IA6 KU-0063794

IA7 Nafamostat

IA8 Pracinostat

IA9 Carbamazepine

IA10 Amiodarone

IA11 MLN 2238

IA12 Blank

IB1 FG-4592

IB2 VX-680

IB3 Quisinostat

IB4 YM155

IB5 CYC 116

IB6 2-Methoxyestradiol

IB7 Omeprazole

IB8 CCT 129202

IB9 Divalproex

IB10 Lacidipine

IB11 MLN 9708

IB12 Blank

IC1 Bortezomib

IC2 Y-27632

IC3 MLN 8054

IC4 Alisertib

IC5 JNJ-26854165

IC6 Temozolomide

IC7 Resveratrol

IC8 SAR 245409

IC9 Gemcitabine

IC10 PCI-34051

IC11 SGI-1776

IC12 Blank

ID1 Erlotinib HCL

ID2 Entinostat

ID3 ZM 447439

ID4 AT 9283

ID5 ENMD-2076

ID6 Vincristine

ID7 Droxinostat

ID8 Hesperadin

ID9 Nimodipine

ID10 Flunarizine

ID11 PP242

ID12 Blank

IE1 PI-103

IE2 Obatoclax

IE3 LY294002

IE4 Barasertib

IE5 Thalidomide

IE6 JNJ-7706621

IE7 Ranolazine

IE8 EX 527

IE9 Azithromycin

IE10 Clevidipine

IE11 TAME

IE12 Blank

IF1 Rapamycin

IF2 Nutlin-3

IF3 Danusertib

IF4 Paclitaxel

IF5 CUDC-101

IF6 WYE-354

IF7 Aurora A

IF8 AZD8055

IF9 Felodipine

IF10 Gabexate

IF11 Degrasyn

IF12 Blank

IG1 Temsirolimus

IG2 Belinostat

IG3 Everolimus

IG4 SNS-314

IG5 Doxurubicin

IG6 Cilnidipine

IG7 PHA-680632

IG8 Fasudil

IG9 Amlodipine

IG10 KW-2449

IG11 (-) Parthenolide

IG12 Blank

IH1 Trichostatin A

IH2 PCI-24781

IH3 Mocetinostat

IH4 CEP-18770

IH5 PFI-1

IH6 Dexamethasone

IH7 MC1568

IH8 Isradipine

IH9 Tamoxifen

IH10 Givinostat

IH11 Chrysophanic

IH12 Blank

**Library AMG-II**

IIA1 Rotundine

IIA2 Tetracaine HCL

IIA3 TAK-901

IIA4 3-Methyladenin

IIA5 Carfilzomib

IIA6 Sodium Phenylbutyrat

IIA7 LDN-57444

IIA8 Piperlongumine

IIA9 GDC-0349

IIA10 Blank

IIA11 Blank

IIA12 Blank

IIB1 Forskolin

IIB2 MG-132

IIB3 AMG-900

IIB4 MK-5108

IIB5 BAY 11-7082

IIB6 Brefeldin

IIB7 TCID

IIB8 GSK 2578215A

IIB9 Scriptaid

IIB10 Blank

IIB11 Blank

IIB12 Blank

IIC1 Bupivacaine HCL

IIC2 PP121

IIC3 Nilvadipine

IIC4 Nocodazole

IIC5 IOX2

IIC6 Oprozomib

IIC7 ONX-0914

IIC8 MHY1485

IIC9 Blank

IIC10 Blank

IIC11 Blank

IIC12 Blank

IID1 Clonidine HCL

IID2 OSI-027

IID3 PF-04691502

IID4 M344

IID5 Pifithrin-α

IID6 PYR-41

IID7 DBeQ

IID8 SBI-0206965

IID9 Blank

IID10 Blank

IID11 Blank

IID12 Blank

IIE1 Loperamide HCL

IIE2 Tubastatin A HCL

IIE3 CCT137690

IIE4 RITA

IIE5 Aspirin

IIE6 PR-619

IIE7 NMS-873

IIE8 Spautin-1

IIE9 Blank

IIE10 Blank

IIE11 Blank

IIE12 Blank

IIF1 Manidipine

IIF2 GSK2126458

IIF3 BGT226

IIF4 Sirtinol

IIF5 Azelnidipine

IIF6 P5091

IIF7 GNE-0877

IIF8 Tenovin-1

IIF9 Blank

IIF10 Blank

IIF11 Blank

IIF12 Blank

IIG1 Manidipine

IIG2 WYE-125132

IIG3 Wortmannin

IIG4 Torin 2

IIG5 Trifluoperazine 2HCL

IIG6 P22077

IIG7 GNE-9605

IIG8 Rocilinostat

IIG9 Blank

IIG10 Blank

IIG11 Blank

IIG12 Blank

IIH1 Nitrendipine

IIH2 Geldanamycin

IIH3 CUDC-907

IIH4 CI994

IIH5 Sulfacetamide Sodium

IIH6 IU1

IIH7 GNE-7915

IIH8 SMI-4a

IIH9 Blank

IIH10 Blank

IIH11 Blank

IIH12 Blank
